# Use of Sutureless and Rapid Deployment Prostheses in Challenging Reoperations

**DOI:** 10.3390/jcdd8070074

**Published:** 2021-06-25

**Authors:** Igor Vendramin, Andrea Lechiancole, Daniela Piani, Gaetano Nucifora, Giovanni Benedetti, Sandro Sponga, Daniele Muser, Uberto Bortolotti, Ugolino Livi

**Affiliations:** 1 Cardiothoracic Department, University Hospital of Udine, 33100 Udine, Italy; vendramin.igor@gmail.com (I.V.); andrea.lechiancole@asufc.sanita.fvg.it (A.L.); daniela.piani@asufc.sanita.fvg.it (D.P.); giovanni.benedetti@asufc.sanita.fvg.it (G.B.); sandro.sponga@asufc.sanita.fvg.it (S.S.); daniele.muser@gmail.com (D.M.); uberto48@gmail.com (U.B.); ugo.livi@asufc.sanita.fvg.it (U.L.); 2 Cardiac Imaging Unit, NorthWest Heart Centre, Manchester University NHS Foundation Trust, Manchester M13 9WL, UK; 3 Department of Medical Area (DAME), University of Udine, 33100 Udine, Italy

**Keywords:** Sutureless valve prosthesis, rapid-deployment valve prosthesis, challenging operations

## Abstract

Sutureless and rapid-deployment bioprostheses have been introduced as alternatives to traditional prosthetic valves to reduce cardiopulmonary and aortic cross-clamp times during aortic valve replacement. These devices have also been employed in extremely demanding surgical settings, as underlined in the present review. Searches on the PubMed and Medline databases aimed to identify, from the English-language literature, the reported cases where both sutureless and rapid-deployment prostheses were employed in challenging surgical situations, usually complex reoperations sometimes even performed as bailout procedures. We have identified 25 patients for whom a sutureless or rapid-deployment prosthesis was used in complex redo procedures: 17 patients with a failing stentless bioprosthesis, 6 patients with a failing homograft, and 2 patients with the failure of a valve-sparing procedure. All patients survived reoperation and were reported to be alive 3 months to 4 years postoperatively. Sutureless and rapid-deployment bioprostheses have proved effective in replacing degenerated stentless bioprostheses and homografts in challenging redo procedures. In these settings, they should be considered as a valid alternative not only to traditional prostheses but also in selected cases to transcatheter valve-in-valve solutions.

## 1. Introduction

Sutureless bioprostheses (SBs) were introduced in clinical practice in the early 2000s [1]; such devices, by avoiding anchoring sutures, were conceived with the aim of shortening the overall surgical and ischemic times during aortic valve replacement (AVR). With the same goal, SBs were subsequently followed by rapid-deployment bioprostheses (RDB), which allow for the reduction of the duration of AVR by using only three guiding sutures tied down after implantation [2]. Currently, one SB, the Perceval S (*LivaNova, Saluggia, Italy*) and one RDB, the Intuity valve system (*Edwards Lifesciences, Irvine, CA, USA*) are available for clinical use, while the first SB produced, the 3f Enable (*Medtronic Inc., Minneapolis, MN, USA*) was withdrawn from the market in 2015 [3].

From the available literature, it appears evident that the initial expectations in terms of the consistent reduction of aortic cross-clamp and total cardiopulmonary bypass (CPB) times have been fully met [4,5]. Moreover, it has also been demonstrated that both SBs and RDBs also provide definite advantages in terms of hemodynamic performance, with a significant reduction in the incidences of severe patient–prosthesis mismatch [6].

Although there are still some concerns about their long-term durability, SBs and RDBs currently represent an appealing alternative not only to traditional bioprostheses but also to transaortic valve implantation (TAVI), as well as in elderly and fragile subjects requiring AVR or reoperations to replace a failing aortic prosthesis—all situations in which the shortening of ischemic times may significantly reduce the operative risks [7,8]. Since SBs and RDBs have shown satisfactory early and medium-term results, they have occasionally also been employed as possible solutions to quite challenging situations, such as complex reoperations, where their use might be still considered ‘off-label’. By analyzing the reported cases, the employment of SBs and RDBs in these scenarios and the results obtained are highlighted in the following review.

## 2. Background

The concept of an SB was pioneered by Magovern and proposed almost 60 years ago to be applied in AVR [9]. In the early years, AVR had a high operative risk mainly due to the prolonged duration of CPB, consequent myocardial ischemia, and suboptimal techniques of myocardial protection. Therefore, a caged-ball prosthesis was devised with the unique feature represented by the possibility of a sutureless implant. The Magovern–Cromie prosthesis was made of a closed stainless steel cage containing a silicone ball; the basal ring contained 9 titanium pins, which, by rotation, could be ejected out and driven into the aorta, securing the device to the aortic annulus. Clinical implants started in 1962, but despite favorable 25-year results, production of this prosthesis ceased in 1980 [10,11]. The sutureless concept has been revitalized today in the most recent models of biological prostheses. Indeed, the reduction of total CPB time is still an important issue, as it appears to be particularly beneficial for fragile, elderly patients referred with increasing frequency for AVR. In fact, in this particular patient subset, the use of tissue valves for AVR has been demonstrated to be advantageous, coupling the benefits of avoidance of chronic anticoagulation and the extended durability of the current generation of bioprostheses [12,13].

## 3. Methods

We have performed an English-language literature search on the PubMed and Medline databases with the aim of identifying cases where both SBs and RDBs were employed in challenging surgical situations—usually complex reoperations sometimes performed even as a bailout procedure. The data were supplemented by those obtained from personal files and charts from the archives of the journals presented on the CTSNet website, and the reference sections of published articles. Articles presenting patient or case series and single case reports were included, but abstracts related to meeting presentations were not considered.

## 4. Results

### 4.1. SB and RDB to Replace a Failing Stentless Bioprosthesis

**Figure 1:**
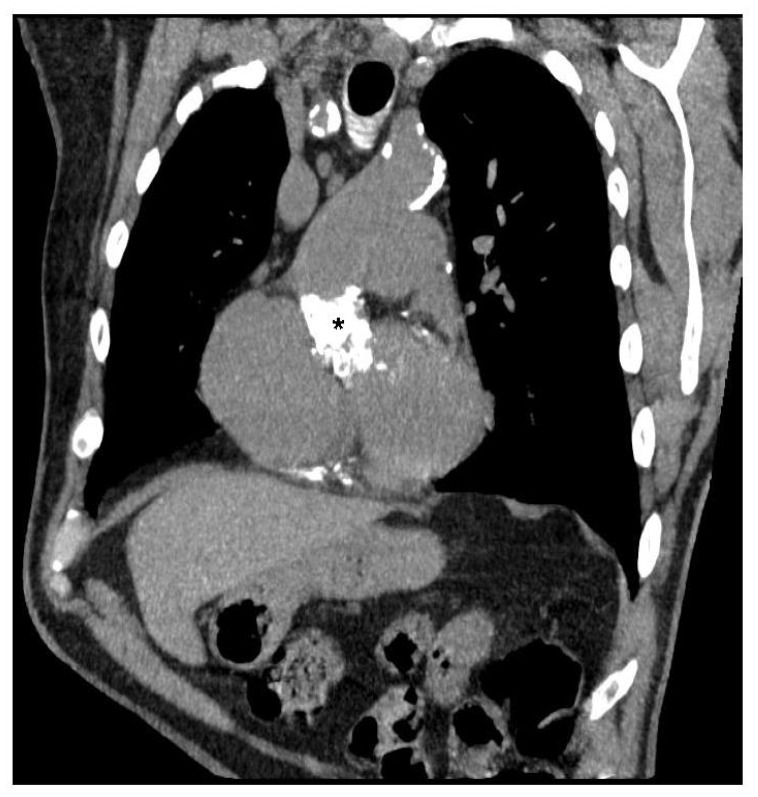
Preoperative computed tomography scan of a 79-year-old man who had undergone a modified Bentall procedure using a 27 mm Prima Plus stentless bioprosthesis, showing the extensively calcified porcine aortic root (*) after 11 years. The porcine aortic valve was replaced with a Perceval M sutureless bioprosthesis.

The data of a total of 17 patients in whom a failing stentless bioprosthesis was replaced with an SB (*n* = 14) [14,15,16,17,18,19,20] or an RDB (*n* = 3) [21,22] were collected. In fact, one patient has been reported about twice [14,19]. There were 11 males and 6 females with an age range of 29 to 84 years at reoperation. A Freestyle porcine aortic root (*Medtronic Inc., Minneapolis, MN, USA*) was employed in 11 cases, a Prima Plus porcine aortic root (*Edwards Lifesciences, Irvine, CA, USA*) in 3 (Figure 1), a Freestyle stentless aortic valve in the subcoronary position in 1, and an Elan stentless aortic valve (*Vascutek Ltd., Inchinnan, UK*) in the subcoronary position in 1; for 1 patient, the type of stentless aortic root implanted was not specified. Failing stentless bioprostheses were replaced with a Perceval SB in 13 cases (size S in 7, M in 2, and L in 4) (Table 1), with an Intuity RDB in 3 (19, 21, and 23 mm) (Table 2) and with a 23 mm 3f Enable SB in 1 (Table 1); 2 patients also required an associated mitral valve repair with a ring annuloplasty [12,13] and one replacement of the ascending aorta [14]. Reoperation was required after 11 to 17 years. Chest reentry was performed through a repeat median sternotomy in 13 cases, while this information was not available for 4 patients. Follow-ups after reoperation ranged from 3 months to 4 years. There were no operative deaths and all patients were reported to be alive at the last follow-up interval, with normally functioning prostheses in echocardiographic controls.

### 4.2. SB and RDB to Replace a Failing Homograft

A failing homograft was replaced in 6 patients using a Perceval SB (*n* = 5, size S) or an Intuity RDB (*n* = 1, 21 mm) (21–26) (Table 3). In 4 patients, the homograft was implanted as a full root replacement, and in 2 as a free-hand AVR. There were 4 males and 2 females with an age range of 39 to 70 years. Reoperation was required 7 to 21 years after the initial homograft implant. For 5 patients, reoperation was performed through a repeat median sternotomy, while for 1, the approach utilized was not indicated. For 2 patients, a combined mitral valve replacement was also performed [23,24]. All patients survived reoperation and 5 were reported to be alive 3 to 44 months postoperatively and with normal prosthetic function, while for one follow-up, the data are not available.

### 4.3. SB to Replace a Failing Aortic Valve Sparing Procedure

This complication has been reported in 2 male patients, 63 and 73 years old, to treat the failure of a valve-sparing operation. In one, reoperation with the implantation of a Perceval XL was required after 3 months. In the other, in whom immediate failure of an aortic valve-sparing procedure occurred intraoperatively with persistent significant aortic regurgitation, a Perceval L was implanted. Both patients were asymptomatic at the 3 and 4-year follow-ups [25,26].

### 4.4. CPB and Aortic Cross-Clamp Times

In the group with failing stentless bioprostheses replaced by an SB, the CPB times were reported in 6 out of 9 patients, ranging from 83 to 223 min (mean of 112 ± 50 min) (10–12,14,16), while the aortic cross-clamp times were reported in 7 out of 9 patients, ranging from 40 to 94 min (mean of 59 ± 17 min) (10–14,16). In one article reporting on 5 patients, the mean CPB and aortic cross-clamp times were 84 ± 4 min and 55 ± 8, respectively [15]. The longest CPB times were recorded when associated procedures were required.

In the group with failing stentless bioprostheses replaced by an RDB, the CPB and ischemic times were reported only in one case, as 55 and 33 min, respectively (17).

In the group undergoing reoperation for failed homografts, CPB times were reported in 4 out of 6 patients (20–23) and aortic cross-clamp times in 5 out of 6 patients (19–23). The CPB times ranged from 61 to 275 min (mean of 127 ± 87 min), and the ischemic time from 34 to 190 min (mean of 72 ± 59 min).

In the 2 patients undergoing reoperation for a failed aortic valve-sparing procedure, the CPB times were 45 and 248 min and the aortic cross-clamp times 26 and 191 min [27,28]. For one case, long times were justified by the need for a second pump run to replace the aortic valve due to persistent regurgitation [28]. 

## 5. Discussion

The recent introduction in the surgical armamentarium of SB clearly demonstrates that old ideas can be effectively turned into modern concepts in the manufacturing of cardiac valve prostheses [29]. SBs have demonstrated in large series and multicenter studies that they provide satisfactory results with low operative mortality, constantly improving outcomes, even when associated procedures are performed, and promising medium-term data [30,31,32]. Similar results have been reported with RDBs with regard to safety, hemodynamic performance, and favorable outcomes [33,34,35,36]. Based on the currently available data, both SBs and RDBs are considered valid alternatives to conventional prostheses for AVR. In particular, when compared to surgical AVR, these devices also allow for shorter CPB and cross-clamp times (4,5). However, SBs and RDBs provide clear-cut advantages beyond the reduction of operative times, such as improved hemodynamic performance and the facilitation of mini-invasive approaches, which renders these devices competitive in specific cases, as well as in comparison with TAVI [3,6,37,38,39].

The present review has underlined that SBs and RDBs are effective, not only for standard AVR but are also extremely useful when dealing with complex surgical scenarios. These are mainly represented by the need to perform redo procedures following previous total aortic root replacement with stentless bioprostheses, or aortic homografts implanted according to a modified Bentall technique. In such patients, after many years, either the porcine aortic root or the aortic wall of the homograft usually becomes heavily calcified, rendering a second Bentall operation extremely hazardous, even if not technically impossible [18,24,40]. Indeed, in such instances, a valve-in-valve procedure has been considered less hazardous when compared to an aortic root re-replacement [41]. In fact, excision of the calcified root and detachment of the coronary buttons may result in injury to such structures, complicating an already cumbersome and demanding procedure. Furthermore, the annulus of the porcine or homograft aortic valve is also often severely calcified, stiff, and narrowed, preventing the positioning of anchoring stitches or a traditional stented prosthesis of adequate size [40]. In these situations, both SBs or RDBs have been proven effective, with no reported operative deaths, even when the procedure was performed as a bailout option [18], and with favorable late outcomes in all cases.

Owing to the limited durability of the first generations of bioprosthetic valves, many centers have acquired considerable experience with reoperation in recipients of degenerated porcine and pericardial bioprostheses [42,43]. It is a general experience that, especially in elective cases, to replace a failing bioprosthesis is, in most cases, not too technically demanding and can be performed with substantially low operative mortality. Despite this, in recent years, the use of TAVI as a valve-in-valve procedure has acquired increasing popularity, particularly owing to its limited invasiveness [44]. This review demonstrates that SBs and RDBs can be used as effectively as TAVI in complex redo procedures, in critically ill subjects, and even in cases where TAVI was considered not technically feasible (18,24). Furthermore, it is noteworthy to consider that SBs have been used for reoperation even in patients with chronic aortic dissection or endocarditis with pseudoaneurysm formation. Occasionally, AVR with an SB has been associated with a graft replacement of the ascending aorta to reconstruct an adequate sino-tubular junction to provide a proper anchoring for the SB cage [16,18,20,45]. A Perceval valve was also employed in combination with a mitral valve replacement or repair, indicating the feasibility and stability of AVR with an SB also after the insertion of a rigid prosthesis or ring in the mitral annulus [17,45,46].

Currently, a mini-invasive approach through a mini-sternotomy or mini-thoracotomy incision is advocated when surgical redo-AVR is planned with an SB or RDB [37,47]. Interestingly, for all patients considered in this review, reoperation was performed through a standard repeat median sternotomy. It is likely, however, that with increasing experience, minimally invasive approaches will also be preferred in complex redo cases using an SB or RDB, although, currently, full sternotomy redo surgery remains the first choice unless specific conditions are present.

An SB was employed in 2 patients to correct the intraoperative failure of a valve-sparing procedure with unsatisfactory repair due to recurrent or intraoperative persistent aortic regurgitation [25,26]. TAVI in the presence of aortic regurgitation is generally still considered an ‘off-label’ procedure and not yet a standard of care in this setting [48,49]. However, the fact that SBs implanted in patients with aortic regurgitation maintained normal function up to 4 years after implantation could stimulate prospective studies aimed to extend the use of percutaneous valves, by using specifically designed devices, to aortic pathologies other than calcific aortic stenosis. 

Besides complex reoperations, and even if beyond the scope of this review, some unusual, often challenging situations, where SBs or RDBs were employed, must also be considered [50]. The Perceval SB has been successfully used for AVR in patients with a porcelain aorta when alternative options, such as a TAVI, were not feasible [51,52,53].

Recently, an Intuity RDB was used in a 59-year-old woman with extensive endocarditis of the aortic valve. The complex repair included the reconstruction of the aortic outflow by closing a large abscess with a double pericardial patch and AVR with an RDB [54]. For this patient, the use of an RDB allowed for shortening a complex procedure, but it should be underlined that in similar settings, favorable results have been obtained with alternative techniques validated on larger patient series, while the effectiveness of the SB in prosthetic valve endocarditis has also been shown [55,56]. 

Stenotic bicuspid aortic valves (BAV) have represented, in the past, a contraindication to SB implants, which, nevertheless, have been used as a Perceval prosthesis in a patient with BAV [57]. In recent years, however, it has been demonstrated that a Perceval SB can be deployed safely in patients with stenotic BAV without increasing the risk of paravalvular leaks, and that BAV should not be currently considered only a relative contraindication to AVR with SB [58]. This has also been confirmed by the data from an international registry demonstrating that the implantation of either an SB or RDB in BAV is more technically demanding but not a contraindication, per se, to the use of such devices [59]. However, important prerequisites for success have been recognized in a detailed analysis of aortic root geometry and with some technical details, particularly the correct decalcification of the aortic annulus and proper sizing [59]. 

## 6. Conclusions

This review has shown that SBs and RDBs represent a clear technological advancement and an important adjunct in prosthetic valve replacement surgery. The current evidence suggests that SBs and RDBs are of great help in extremely challenging situations, such as complex reoperations, particularly when undertaken for stentless valves or homografts failures. In these settings, such devices allow for a limited surgical approach, avoiding complex aortic root re-replacement and significantly reducing the risk of reoperation. Based on the results of the present review, and once evidence is provided on consistent medium and long-term durability, it will be possible to consider unusual or even ‘off-label’ employment of both SBs and RDBs in future recommendations.

## Figures and Tables

**Table 1:** Use of sutureless bioprosthesis in challenging reoperations. Summary of reported cases.

Author	Year	Age, Sex	Operation	Reoperation	Outcome, FU
Villa et al. ^10^	2013	63, F	21 mm Freestyle aortic root	Perceval S	Alive, NA
Kim et al. ^11^	2015	78, M	25 mm Prima aortic root	Perceval S	Alive, 3 months
Lio et al. ^12^	2016	72, F	25 mm Freestyle aortic root	Perceval S, MVr	Alive, 7 months
		83, F	27 mm Freestyle aortic root	Perceval L	Alive, 1 year
Götte et al. ^13^	2016	83, M	25 mm stentless aortic root *	Perceval M, MVr	Alive, NA
Marzouk et al. ^14^	2016	78, M	23 mm Freestyle subcoronary, AAR	Perceval S	Alive, 6 months
Chiariello et al. ^15^	2017	3M, 2F ** Mean age, 69 ± 6 years	21 mm Freestyle aortic root (*n* = 1); 27 mm Freestyle aortic root (*n* = 2); 23 mm Prima aortic rot (*n* = 1); 25 mm Prima aortic root (*n* = 1)	Perceval S (*n* = 2), M (*n* = 1), L (*n* = 2)	Mean, 27 ± 16 months
Stoker et al. ^16^	2018	44, F	21 mm Freestyle aortic root	Perceval S	Alive, NA
		29, M	27 mm Freestyle aortic root	Perceval L	Alive, NA
		76, M	23 mm Freestyle aortic root	23 mm Enable	Alive, NA

FU = Follow-up; NA = Not available; S = Small; M = Medium; L = Large; MVr = Mitral valve repair; AAR = Ascending aorta replacement. * The Stentless bioprosthesis model is not specified; ** One of these patients was previously reported (see reference #10), and therefore, was excluded from the table. Numeric superscript: reported cases number.

**Table 2:** Use the Intuity rapid deployment bioprosthesis in challenging reoperations. Summary of reported cases.

Author	Year	Age, Sex	Operation	Reoperation	Outcome, FU
Gariboldi et al. ^19^	2013	50, M	Freestyle aortic root	Intuity 21 mm	Alive, NA
Martinelli et al. ^20^	2015	69, M	Freestyle 25 mm aortic root	Intuity 23 mm	Alive, 6 months
		84, F	Elan SB subcoronary	Intuity 19 mm	Alive, 6 months

FU = Follow-up; NA = Not available; SB = Stentless bioprosthesis. Numeric superscript: reported cases number.

**Table 3:** Reoperation for failed homografts using Perceval of Intuity bioprostheses. Summary of reported cases.

Author	Year	Age, Sex	Operation	Reoperation	Outcome, FU
Folliguet et al. ^21^	2013	62, M	23 mm homograft aortic root	Perceval S	Alive, 6 months
Čanádyová et al. ^22^	2015	70, F	AVR, freehand homograft	Perceval S	Alive, NA
Dohmen et al. ^23^	2016	61, F	21 mm homograft aortic root	Perceval S	Alive, 1 year
Folesani et al. ^24^	2016	50, M	AVR, 24 mm freehand homograft	21 mm Intuity	Alive, 6 months
Akca et al. ^25^	2017	55, M	Homograft aortic root	Perceval S, MVR	Alive, 3 months
Hammond et al. ^26^	2020	39, M	Homograft aortic root	Perceval S, MVR	Alive, 44 months

FU = Follow-up; S = Small; AVR = Aortic valve replacement; NA = Not available; MVR = Mitral valve replacement. Numeric superscript: reported cases number.
